# 3-[2-(2,3-Dioxoindolin-1-yl)eth­yl]-1,3-oxazolidin-2-one

**DOI:** 10.1107/S1600536810002576

**Published:** 2010-01-27

**Authors:** Abdusalam Al Subari, Rachid Bouhfid, Hafid Zouihri, El Mokhtar Essassi, Seik Weng Ng

**Affiliations:** a Laboratoire de Chimie Organique Hétérocyclique, Pôle de Compétences Pharmacochimie, Université Mohammed V-Agdal, BP 1014 Avenue Ibn Batout, Rabat, Morocco; b Institute of Nanomaterials and Nanotechnology, Avenue de l’Armée Royale, Madinat El Irfane, 10100 Rabat, Morocco; c CNRST Division of UATRS Angle Allal Fassi/FAR, BP 8027 Hay Riad, 10000 Rabat, Morocco; d Department of Chemistry, University of Malaya, 50603 Kuala Lumpur, Malaysia

## Abstract

In the title compound, C_13_H_12_N_2_O_4_, the almost planar (r.m.s. deviation = 0.012 Å) dioxoindolinyl unit and the envelope-shaped oxazolidine ring (with the methyl­ene C atom bonded to the N atom as the flap) are linked by a —CH_2_—CH_2_— bridge, in which the N—C—C—N unit adopts a *gauche* conformation [torsion angle = 62.7 (2)°].

## Related literature

For the synthesis of compounds with dioxoindolinyl and oxazolidinyl units, see: Alsubari *et al.* (2009 ▶); Bouhfid *et al.* (2008 ▶).
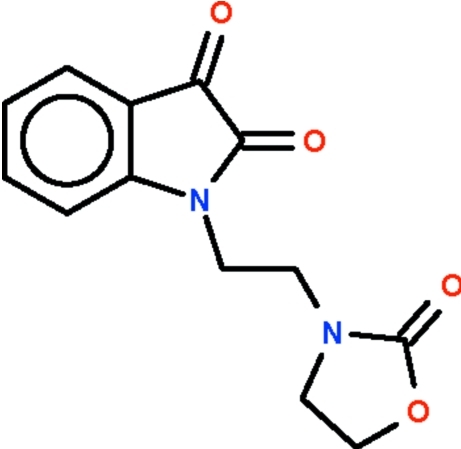


## Experimental

### 

#### Crystal data

C_13_H_12_N_2_O_4_
*M* *_r_* = 260.25
Triclinic, 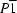

*a* = 7.1198 (2) Å
*b* = 7.4694 (2) Å
*c* = 12.0319 (3) Å
α = 83.338 (2)°
β = 79.084 (2)°
γ = 81.372 (2)°
*V* = 618.64 (3) Å^3^
*Z* = 2
Mo *K*α radiation
μ = 0.11 mm^−1^
*T* = 293 K
0.3 × 0.3 × 0.3 mm

#### Data collection

Bruker APEXII diffractometer
16105 measured reflections
2856 independent reflections
2105 reflections with *I* > 2σ(*I*)
*R*_int_ = 0.029

#### Refinement

*R*[*F* ^2^ > 2σ(*F* ^2^)] = 0.037
*wR*(*F* ^2^) = 0.129
*S* = 1.07
2856 reflections
172 parameters
H-atom parameters constrained
Δρ_max_ = 0.15 e Å^−3^
Δρ_min_ = −0.22 e Å^−3^

### 

Data collection: *APEX2* (Bruker, 2005 ▶); cell refinement: *SAINT* (Bruker, 2005 ▶); data reduction: *SAINT*; program(s) used to solve structure: *SHELXS97* (Sheldrick, 2008 ▶); program(s) used to refine structure: *SHELXL97* (Sheldrick, 2008 ▶); molecular graphics: *X-SEED* (Barbour, 2001 ▶); software used to prepare material for publication: *publCIF* (Westrip, 2010 ▶).

## Supplementary Material

Crystal structure: contains datablocks global, I. DOI: 10.1107/S1600536810002576/bt5178sup1.cif

Structure factors: contains datablocks I. DOI: 10.1107/S1600536810002576/bt5178Isup2.hkl

Additional supplementary materials:  crystallographic information; 3D view; checkCIF report

## Figures and Tables

**Fig. 1.**
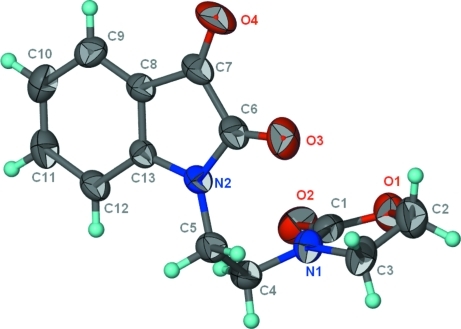
Anisotropic displacement ellipsoid plot (Barbour, 2001) of C13H12N2O4 at the 50% probability level; hydrogen atoms are drawn as spheres of an arbitrary radius.

## supplementary crystallographic information

### Experimental

Indoline-2, 3-dione (1 g, 6.8 mmol), bis(chloroethyl)amine (0.96 g, 6.8 mmol) and potassium carbonate (1 g, 7.2 mmol) along with catalytic amount of tetra-*n*-butylammonium bromide were stirred in DMF (30 ml) for 72 h. After the completion of the reaction (as monitored by TLC) , the solid material was removed by filtration and the solvent evaporated under vacuum. Dichloromethane (20 ml) was added and the solution filtered. The solvent was removed and the product purified byrecrystallization from ethanol to afford red crystals in 60% yield . The formulation of the product was established by proton and carbon-13 NMR spectroscopy.

### Refinement

H-atoms were placed in calculated positions (C—H 0.93–0.97 Å) and were included in the refinement in the riding model approximation, with *U*(H) set to 1.2–1.5*U*(C).

### Crystal data

**Table:**

C_13_H_12_N_2_O_4_	*Z* = 2
*M**_r_* = 260.25	*F*(000) = 272
Triclinic, *P*1	*D*_x_ = 1.397 Mg m^−^^3^
Hall symbol: -P 1	Mo *K*α radiation, λ = 0.71073 Å
*a* = 7.1198 (2) Å	Cell parameters from 4652 reflections
*b* = 7.4694 (2) Å	θ = 2.7–25.5°
*c* = 12.0319 (3) Å	µ = 0.11 mm^−^^1^
α = 83.338 (2)°	*T* = 293 K
β = 79.084 (2)°	Block, red
γ = 81.372 (2)°	0.3 × 0.3 × 0.3 mm
*V* = 618.64 (3) Å^3^	

### Data collection

**Table:**

Bruker APEXII diffractometer	2105 reflections with *I* > 2σ(*I*)
Radiation source: fine-focus sealed tube	*R*_int_ = 0.029
graphite	θ_max_ = 27.5°, θ_min_ = 1.7°
φ and ω scans	*h* = −9→9
16105 measured reflections	*k* = −9→9
2856 independent reflections	*l* = −15→15

### Refinement

**Table:**

Refinement on *F*^2^	Primary atom site location: structure-invariant direct methods
Least-squares matrix: full	Secondary atom site location: difference Fourier map
*R*[*F*^2^ > 2σ(*F*^2^)] = 0.037	Hydrogen site location: inferred from neighbouring sites
*wR*(*F*^2^) = 0.129	H-atom parameters constrained
*S* = 1.07	*w* = 1/[σ^2^(*F*_o_^2^) + (0.0678*P*)^2^ + 0.0776*P*] where *P* = (*F*_o_^2^ + 2*F*_c_^2^)/3
2856 reflections	(Δ/σ)_max_ = 0.001
172 parameters	Δρ_max_ = 0.15 e Å^−^^3^
0 restraints	Δρ_min_ = −0.21 e Å^−^^3^

### Fractional atomic coordinates and isotropic or equivalent isotropic displacement parameters (Å^2^)

**Table:**

	*x*	*y*	*z*	*U*_iso_*/*U*_eq_	
O1	0.52252 (19)	0.72705 (19)	0.96655 (11)	0.0698 (4)	
O2	0.62176 (17)	0.59350 (18)	0.80356 (10)	0.0650 (4)	
O3	0.0398 (2)	1.06952 (18)	0.81418 (11)	0.0710 (4)	
O4	0.1729 (2)	1.32239 (15)	0.61954 (12)	0.0735 (4)	
N1	0.30085 (18)	0.66870 (17)	0.87600 (10)	0.0465 (3)	
N2	0.13257 (17)	0.86176 (15)	0.67978 (10)	0.0409 (3)	
C1	0.4910 (2)	0.6559 (2)	0.87395 (13)	0.0485 (4)	
C2	0.3389 (3)	0.8000 (3)	1.02957 (17)	0.0723 (5)	
H2A	0.3186	0.9316	1.0167	0.087*	
H2B	0.3325	0.7662	1.1104	0.087*	
C3	0.1916 (3)	0.7179 (3)	0.98519 (14)	0.0599 (4)	
H3A	0.1523	0.6121	1.0337	0.072*	
H3B	0.0788	0.8053	0.9766	0.072*	
C4	0.2235 (2)	0.5789 (2)	0.79784 (13)	0.0472 (4)	
H4A	0.1725	0.4711	0.8374	0.057*	
H4B	0.3267	0.5405	0.7369	0.057*	
C5	0.0649 (2)	0.7017 (2)	0.74742 (13)	0.0470 (4)	
H5A	0.0116	0.6330	0.7000	0.056*	
H5B	−0.0378	0.7404	0.8085	0.056*	
C6	0.1067 (2)	1.0296 (2)	0.71898 (13)	0.0477 (4)	
C7	0.1775 (2)	1.16148 (19)	0.61515 (14)	0.0482 (4)	
C8	0.2403 (2)	1.04883 (18)	0.52062 (12)	0.0408 (3)	
C9	0.3139 (2)	1.0915 (2)	0.40725 (14)	0.0520 (4)	
H9	0.3330	1.2101	0.3797	0.062*	
C10	0.3580 (3)	0.9532 (3)	0.33635 (14)	0.0588 (4)	
H10	0.4078	0.9783	0.2598	0.071*	
C11	0.3288 (2)	0.7779 (2)	0.37813 (14)	0.0563 (4)	
H11	0.3589	0.6870	0.3285	0.068*	
C12	0.2556 (2)	0.7318 (2)	0.49236 (13)	0.0464 (4)	
H12	0.2377	0.6128	0.5198	0.056*	
C13	0.21104 (18)	0.87131 (18)	0.56258 (11)	0.0368 (3)	

### Atomic displacement parameters (Å^2^)

**Table:**

	*U*^11^	*U*^22^	*U*^33^	*U*^12^	*U*^13^	*U*^23^
O1	0.0697 (8)	0.0806 (9)	0.0668 (8)	−0.0181 (7)	−0.0215 (6)	−0.0121 (7)
O2	0.0485 (7)	0.0773 (8)	0.0623 (8)	−0.0093 (6)	0.0045 (6)	0.0006 (6)
O3	0.0900 (9)	0.0660 (8)	0.0544 (7)	0.0057 (7)	−0.0051 (6)	−0.0280 (6)
O4	0.0969 (10)	0.0341 (6)	0.0964 (10)	−0.0070 (6)	−0.0291 (8)	−0.0164 (6)
N1	0.0461 (7)	0.0516 (7)	0.0404 (7)	−0.0029 (5)	−0.0048 (5)	−0.0079 (5)
N2	0.0463 (7)	0.0356 (6)	0.0416 (6)	−0.0052 (5)	−0.0067 (5)	−0.0086 (5)
C1	0.0532 (9)	0.0461 (8)	0.0448 (8)	−0.0110 (7)	−0.0066 (7)	0.0045 (6)
C2	0.0919 (15)	0.0705 (12)	0.0573 (11)	−0.0044 (10)	−0.0156 (10)	−0.0205 (9)
C3	0.0624 (11)	0.0717 (11)	0.0414 (9)	−0.0004 (8)	−0.0018 (7)	−0.0108 (8)
C4	0.0548 (9)	0.0405 (7)	0.0456 (8)	−0.0087 (6)	−0.0054 (7)	−0.0041 (6)
C5	0.0440 (8)	0.0493 (8)	0.0484 (8)	−0.0124 (6)	−0.0045 (6)	−0.0052 (7)
C6	0.0503 (8)	0.0427 (8)	0.0515 (9)	0.0020 (6)	−0.0125 (7)	−0.0152 (6)
C7	0.0504 (8)	0.0358 (7)	0.0633 (10)	−0.0014 (6)	−0.0220 (7)	−0.0107 (6)
C8	0.0405 (7)	0.0354 (7)	0.0496 (8)	−0.0031 (5)	−0.0170 (6)	−0.0048 (6)
C9	0.0538 (9)	0.0514 (9)	0.0524 (9)	−0.0100 (7)	−0.0186 (7)	0.0082 (7)
C10	0.0612 (10)	0.0749 (12)	0.0403 (9)	−0.0089 (8)	−0.0113 (7)	−0.0011 (8)
C11	0.0608 (10)	0.0628 (10)	0.0471 (9)	0.0019 (8)	−0.0124 (7)	−0.0205 (8)
C12	0.0531 (9)	0.0374 (7)	0.0505 (9)	−0.0023 (6)	−0.0118 (7)	−0.0123 (6)
C13	0.0354 (7)	0.0358 (7)	0.0413 (7)	−0.0026 (5)	−0.0119 (5)	−0.0070 (5)

### Geometric parameters (Å, °)

**Table:**

O1—C1	1.357 (2)	C4—H4A	0.9700
O1—C2	1.445 (2)	C4—H4B	0.9700
O2—C1	1.2099 (19)	C5—H5A	0.9700
O3—C6	1.2072 (18)	C5—H5B	0.9700
O4—C7	1.2043 (18)	C6—C7	1.552 (2)
N1—C1	1.339 (2)	C7—C8	1.455 (2)
N1—C4	1.4458 (19)	C8—C9	1.384 (2)
N1—C3	1.4509 (19)	C8—C13	1.394 (2)
N2—C6	1.3652 (18)	C9—C10	1.378 (2)
N2—C13	1.4127 (17)	C9—H9	0.9300
N2—C5	1.4570 (19)	C10—C11	1.378 (3)
C2—C3	1.497 (3)	C10—H10	0.9300
C2—H2A	0.9700	C11—C12	1.397 (2)
C2—H2B	0.9700	C11—H11	0.9300
C3—H3A	0.9700	C12—C13	1.3799 (19)
C3—H3B	0.9700	C12—H12	0.9300
C4—C5	1.518 (2)		
			
C1—O1—C2	108.70 (14)	N2—C5—H5A	109.0
C1—N1—C4	121.55 (12)	C4—C5—H5A	109.0
C1—N1—C3	111.44 (13)	N2—C5—H5B	109.0
C4—N1—C3	123.13 (13)	C4—C5—H5B	109.0
C6—N2—C13	110.57 (12)	H5A—C5—H5B	107.8
C6—N2—C5	123.66 (13)	O3—C6—N2	127.54 (16)
C13—N2—C5	125.33 (11)	O3—C6—C7	126.54 (14)
O2—C1—N1	128.43 (15)	N2—C6—C7	105.91 (12)
O2—C1—O1	122.25 (16)	O4—C7—C8	131.16 (17)
N1—C1—O1	109.31 (14)	O4—C7—C6	123.49 (15)
O1—C2—C3	105.01 (14)	C8—C7—C6	105.34 (12)
O1—C2—H2A	110.7	C9—C8—C13	121.28 (13)
C3—C2—H2A	110.7	C9—C8—C7	131.54 (14)
O1—C2—H2B	110.7	C13—C8—C7	107.17 (13)
C3—C2—H2B	110.7	C10—C9—C8	118.13 (15)
H2A—C2—H2B	108.8	C10—C9—H9	120.9
N1—C3—C2	100.50 (14)	C8—C9—H9	120.9
N1—C3—H3A	111.7	C11—C10—C9	120.42 (15)
C2—C3—H3A	111.7	C11—C10—H10	119.8
N1—C3—H3B	111.7	C9—C10—H10	119.8
C2—C3—H3B	111.7	C10—C11—C12	122.36 (15)
H3A—C3—H3B	109.4	C10—C11—H11	118.8
N1—C4—C5	112.13 (12)	C12—C11—H11	118.8
N1—C4—H4A	109.2	C13—C12—C11	116.79 (14)
C5—C4—H4A	109.2	C13—C12—H12	121.6
N1—C4—H4B	109.2	C11—C12—H12	121.6
C5—C4—H4B	109.2	C12—C13—C8	121.01 (13)
H4A—C4—H4B	107.9	C12—C13—N2	127.98 (13)
N2—C5—C4	112.80 (12)	C8—C13—N2	111.01 (11)
			
C4—N1—C1—O2	10.0 (2)	O3—C6—C7—C8	−178.75 (15)
C3—N1—C1—O2	168.48 (16)	N2—C6—C7—C8	0.25 (15)
C4—N1—C1—O1	−170.25 (13)	O4—C7—C8—C9	0.1 (3)
C3—N1—C1—O1	−11.80 (18)	C6—C7—C8—C9	178.84 (14)
C2—O1—C1—O2	176.40 (16)	O4—C7—C8—C13	−178.82 (17)
C2—O1—C1—N1	−3.34 (19)	C6—C7—C8—C13	−0.11 (15)
C1—O1—C2—C3	16.2 (2)	C13—C8—C9—C10	−0.1 (2)
C1—N1—C3—C2	20.76 (19)	C7—C8—C9—C10	−178.89 (15)
C4—N1—C3—C2	178.81 (14)	C8—C9—C10—C11	0.1 (2)
O1—C2—C3—N1	−21.31 (19)	C9—C10—C11—C12	−0.4 (3)
C1—N1—C4—C5	−135.55 (14)	C10—C11—C12—C13	0.7 (2)
C3—N1—C4—C5	68.55 (18)	C11—C12—C13—C8	−0.6 (2)
C6—N2—C5—C4	−99.82 (16)	C11—C12—C13—N2	178.77 (13)
C13—N2—C5—C4	88.45 (16)	C9—C8—C13—C12	0.3 (2)
N1—C4—C5—N2	62.71 (16)	C7—C8—C13—C12	179.42 (13)
C13—N2—C6—O3	178.69 (15)	C9—C8—C13—N2	−179.15 (12)
C5—N2—C6—O3	5.9 (2)	C7—C8—C13—N2	−0.07 (15)
C13—N2—C6—C7	−0.30 (15)	C6—N2—C13—C12	−179.21 (14)
C5—N2—C6—C7	−173.10 (12)	C5—N2—C13—C12	−6.6 (2)
O3—C6—C7—O4	0.1 (3)	C6—N2—C13—C8	0.24 (16)
N2—C6—C7—O4	179.09 (15)	C5—N2—C13—C8	172.90 (12)
